# Hydroxychloroquine effectiveness in reducing symptoms of hand osteoarthritis (HERO): study protocol for a randomized controlled trial

**DOI:** 10.1186/1745-6215-14-64

**Published:** 2013-03-02

**Authors:** Sarah R Kingsbury, Puvan Tharmanathan, Joy Adamson, Nigel K Arden, Fraser Birrell, Sarah Cockayne, John Dickson, Michael Doherty, Krysia S Dziedzic, Andrew Grainger, Catherine E Hewitt, Terence W O’Neill, David L Scott, Tonia L Vincent, Richard J Wakefield, Fiona E Watt, David J Torgerson, Philip G Conaghan

**Affiliations:** 1 Division of Rheumatic and Musculoskeletal Disease and National Institute of Health Research (NIHR) Leeds Musculoskeletal Biomedical Research Unit, University of Leeds, Leeds LS7 4SA, UK; 2 York Trials Unit, Department of Health Sciences, University of York, York, UK; 3 Medical Research Council (MRC) Lifecourse Epidemiology Unit, University of Southampton, Southampton, UK and NIHR Musculoskeletal Biomedical Research Unit, Nuffield Department of Orthopaedics, Rheumatology and Musculoskeletal Sciences, Nuffield Orthopaedic Centre, University of Oxford, Oxford, UK; 4 Rheumatology, Northumbria Healthcare NHS Foundation, Ashington, Northumberland, UK and Musculoskeletal Research Group, Institute of Cellular Medicine, Newcastle University, Newcastle, UK; 5 South Tees Hospitals NHS Foundation Trust, Middlesbrough, Redcar and Cleveland Specialist Musculoskeletal Service, Cleveland, UK; 6 Academic Rheumatology, University of Nottingham, Nottingham, UK; 7 Arthritis Research UK Primary Care Centre, Keele University, Keele, UK; 8 Arthritis Research UK Epidemiology Unit, University of Manchester, Manchester, UK; 9 Department of Rheumatology, King’s College London School of Medicine, King’s College London and Department of Rheumatology, King’s College Hospital, London, UK; 10 Kennedy Institute of Rheumatology, Nuffield Department of Orthopaedics, Rheumatology and Musculoskeletal Sciences, University of Oxford, UK and Imperial College London, London, UK

**Keywords:** Double-blind, Hand osteoarthritis, Hydroxychloroquine, Placebo-controlled, Randomized

## Abstract

**Background:**

Osteoarthritis (OA) is the most common type of arthritis, causing significant joint pain and disability. It is already a major cause of healthcare expenditure and its incidence will further increase with the ageing population. Current treatments for OA have major limitations and new analgesic treatments are needed. Synovitis is prevalent in OA and is associated with pain. Hydroxychloroquine is used in routine practice for treating synovitis in inflammatory arthritides, such as rheumatoid arthritis. We propose that treating patients with symptomatic hand OA with hydroxychloroquine will be a practical and safe treatment to reduce synovitis and pain.

**Methods/design:**

HERO is an investigator-initiated, multicentre, randomized, double-blind, placebo-controlled trial. A total of 252 subjects with symptomatic hand OA will be recruited across primary and secondary care sites in the UK and randomized on a 1:1 basis to active treatment or placebo for 12 months. Daily medication dose will range from 200 to 400 mg according to ideal body weight. The primary endpoint is change in average hand pain during the previous two weeks (measured on a numerical rating scale (NRS)) between baseline and six months. Secondary endpoints include other self-reported pain, function and quality-of-life measures and radiographic structural change at 12 months. A health economics analysis will also be performed. An ultrasound substudy will be conducted to examine baseline levels of synovitis. Linear and logistic regression will be used to compare changes between groups using univariable and multivariable modelling analyses. All analyses will be conducted on an intention-to-treat basis.

**Discussion:**

The HERO trial is designed to examine whether hydroxychloroquine is an effective analgesic treatment for OA and whether it provides any long-term structural benefit. The ultrasound substudy will address whether baseline synovitis is a predictor of therapeutic response. This will potentially provide a new treatment for OA, which could be of particular use in the primary care setting.

**Trial registration:**

ISRCTN91859104.

## Background

Osteoarthritis (OA) is an increasingly common problem in ageing Western societies, affecting an estimated 8.5 million people in the UK and causing both an enormous burden to health systems and considerable pain and disability to affected individuals [1,2]. Although radiographic hand OA (RHOA) is recognized as being highly prevalent in the older population, with 60 to 70% of people over the age of 55 estimated to have RHOA [3], there is a common misconception that symptomatic hand OA is not a prevalent disease. However population data suggests symptomatic hand OA to be more prevalent than symptomatic knee OA [4], affecting 8% of people aged 60 or over [5] and 26% of women and 13% of men aged 70 or over [6]. The notable paucity of published clinical research examining the clinical impact, epidemiology and therapy of hand OA, as compared with hip and knee OA [7], may be attributed to patients with symptomatic hand OA failing to seek medical care [8], perhaps because of both their own perception of a lack of treatment options and reinforcement of this view by medical practitioners [9].

The most commonly involved joints in symptomatic hand OA are the distal and proximal interphalangeal joints, followed by the base of the thumb [6]. Significant difficulties with daily tasks, such as gripping, writing, carrying heavy items and picking up small objects, have been reported by patients with symptomatic hand OA [6], with 50% reporting problems in wringing out washcloths and opening jars and a 60% reduction in grip strength [10]. Moreover, the onset of hand OA significantly impacts on the deterioration of global physical functioning, irrespective of concurrent lower-limb joint pain [11]. Symptomatic hand OA, therefore, represents a considerable economic, clinical and social burden.

Current guidelines for hand OA treatment, such as those from the UK National Institute for Health and Clinical Excellence (NICE) and the European League against Rheumatism (EULAR), recommend topical treatments, such as NSAID gel and capsaicin cream, oral analgesia (including paracetamol and oral NSAIDs) and nonpharmacological therapy [12,13]. However, these treatments are precluded by comorbidities and often restricted in their duration by degree of efficacy and considerable associated toxicities. Moreover, since the majority of data guiding treatment for OA is derived from studies on the knee, more studies on therapies for symptomatic hand OA are required.

Although traditionally considered a disease of articular cartilage, modern imaging studies have vastly improved our understanding of the complex inter-relationship of the tissues involved in the pathophysiology of OA, and clearly demonstrate OA to be a disease of the whole joint, involving subchondral bone changes, osteophyte formation and synovial inflammation [14,15]. In knee OA, a change in MRI-detected synovitis score is significantly correlated with a change in knee-pain score (*P* < 0.001, *r* = 0.21) [16,17], while 82% of painful OA hand joints had ultrasound-detected synovitis, with painful hand joints more likely to have synovitis than nonpainful hand joints (*P* < 0.001) [18]. Moreover, 86% of patients with American College of Rheumatology (ACR) hand OA and erosive changes seen on X-ray displayed ultrasound-detected synovial thickening and 82% an increased power Doppler signal [19]. Patients with higher levels of ultrasound-detected synovitis at baseline have also been shown to have a better response to intramuscular steroids, which are thought to work by reducing synovitis [20]. Taken together, these studies suggest that treatments to target synovitis may be effective in reducing pain in OA.

Hydroxychloroquine has been successfully used for many years in the treatment of inflammatory arthritides, such as rheumatoid arthritis (RA) and systemic lupus erythematosus, and less commonly in the seronegative spondyloarthropathies [21,22]. Placebo-controlled trials in RA have demonstrated significant efficacy of hydroxychloroquine, both as a monotherapy and in combination with other RA drugs, and - due to its excellent safety profile it remains a popular therapy for RA. Although hydroxychloroquine’s mechanism of action in RA is poorly understood, it is presumed to be associated with an anti-synovial activity.

**Figure 1:**
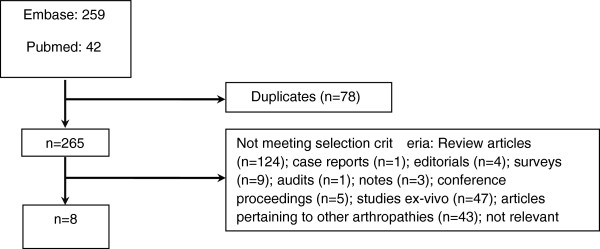
**Overview of systematic review of hydroxychloroquine use in osteoarthritis.** Databases: PubMed, MEDLINE and Embase. Search terms: MeSH headings #1 ‘osteoarthritis’ and #2 ‘hydroxychloroquine’ or ‘chloroquine’. Limits: Humans.

Although hydroxychloroquine has been used on an anecdotal basis for the treatment of OA, there have been few studies to assess its efficacy and these studies have contained only small numbers of patients. A summary of these studies is presented in Figure 1 and Table 1. The small numbers of patients, different inclusion criteria and outcome measures used in these trials allow limited conclusions to be drawn. However, the reported improvement in patient symptoms compared with control arms in the majority of these studies suggests a need for a properly designed and well-powered trial to be performed.

**Table 1:** Systematic review of hydroxychloroquine use in osteoarthritis

**Reference**	***n***	**Site**	**Treatment**	**Outcome**
[23]	8	Erosive hand OA unresponsive to NSAIDs	200 mg HCQ	6/8 noted improvement in pain, reduced synovitis and reduced morning stiffness
				Response time 7/52 to 7/12. No adverse effects
[24]	15	Hand OA	Placebo-controlled HCQ	Improvement in clinical symptoms at 12 months (Ritchie index)
[25]	7	Erosive hand OA	200 to 400 mg HCG	Improvement in 7/7 patients noted
[26]	15	Hand and knee OA	HCQ 6/12	13/15 patients achieved good therapeutic response after 6 months
[27]	29	Knee OA	400 mg HCQ 4/12, placebo-controlled	No difference in WOMAC pain (*P* = 0.551), stiffness (*P* = 0.512) or function (*P* = 0.293); no difference on VAS (*P* = 0.461) or Lequesne (*P* = 0.803) scales
[28]	88	Nodal OA of the hands	HCQ 200 mg/bid, ACM 1.3 gm/tid or placebo 6/12	No significant difference between hydroxychloroquine, acetaminophen or placebo in mean number of tender joints at 24 weeks
[29]	8	Erosive or inflammatory OA	HCQ 200 to 400 mg	Clinical improvement in 5/8 patients, 3 patients discontinued (1 due to unresponsiveness, 2 due to side-effects)
[30]				Intra-articular chloroquine in RA and OA of knee joint
				No abstract available

ACM, acetaminophen; HCQ, hydroxychloroquine; MeSH, medical subject headings; VAS, visual analogue scale; WOMAC, Western Ontario and McMaster Universities Index of OA.

We propose that treating patients with symptomatic hand OA with hydroxychloroquine will be a practical and safe treatment to reduce synovitis and therefore reduce pain. We believe that the preliminary data from the handful of small studies previously conducted and anecdotal evidence of hydroxychloroquine efficacy in hand OA strongly support the need for a well-designed, adequately powered, randomized placebo-controlled trial to examine fully the potential use of hydroxychloroquine as a treatment for OA. The hydroxychloroquine effectiveness in reducing symptoms of hand osteoarthritis (HERO) trial was designed to this end. The protocol of the HERO trial is outlined in this paper.

## Methods/design

### Trial development

The trial was designed with key stakeholders including rheumatologists with experience of treating OA, general practitioners with a special interest in musculoskeletal disease, methodologists and users with experience of hand OA.

### Trial objectives

The trial will aim to address the following questions:

1. Is hydroxychloroquine an effective analgesic treatment for OA?

2. What is the time course for symptomatic relief with hydroxychloroquine?

3. Does hydroxychloroquine treatment provide any long-term structural benefit?

The ultrasound substudy will address whether baseline synovitis is a predictor of therapeutic response.

### Population

All adults with symptomatic, radiographic hand osteoarthritis and inadequate response or toxicity to their existing medication (to include paracetamol, oral NSAID or opioid).

### Intervention

Hydroxychloroquine is a 4-aminoquinoline anti-malarial drug that, because of its weak diprotic base properties, is able to pass through lipid cell membranes and preferentially accumulate in acidic cytoplasmic vesicles within macrophages and antigen-presenting cells. *In vitro* studies demonstrate that by increasing vesicle pH, hydroxychloroquine is able to modulate the antigen-processing activity of these cells, resulting in down-regulation of the immune response [31]. Moreover, hydroxychloroquine is able to block T-cell activation [32] and reduce the release of various cytokines, including interleukin (IL)-1, IL-6, tumour necrosis factor and IL-1β-induced nitric oxide, that have been demonstrated to be involved in OA inflammation and cartilage degeneration [33-35]. It has also significantly reduced matrix metalloprotease levels in a rat calcium pyrophosphate disease model [36]. Inhibition of cytokine production and reduction of T-cell activity is the probable mechanism underlying hydroxychloroquine’s efficacy in RA. The relevance of these inflammatory pathways to OA pathology [21,22,31,32], coupled with the evidence that synovitis is closely correlated with pain in the OA joint, suggests that hydroxychloroquine might also be an efficacious analgesic agent for the treatment of OA.

### Outcome measures

The HERO trial will examine a range of clinical, imaging and quality-of-life and economic outcomes, in line with the objectives listed.

### Clinical outcome measures

The primary endpoint of the study will be change in ‘average overall hand pain severity over the previous two weeks’ (as graded on a 0 to 10 numerical rating scale) between baseline and six months. Secondary outcome measures are outlined in Table 2.

**Table 2:** Outcome measures and timetable

	**Month(s)**			
	**0**	**3**	**6**	**12**
**Primary outcome**				
Average overall hand pain severity over the previous 2 weeks (0 to 10 NRS)	✓	✓	✓	✓
**Secondary outcomes**				
Imaging assessments				
• Bilateral hand X-ray	✓			✓
• Ultrasound synovitis score	✓			
Clinical assessments				
• Grip strength	✓		✓	✓
• Joint count	✓		✓	✓
Self-reported questionnaires				
• AUSCAN [37,38] (pain, stiffness and function) – five-point Likert scale	✓	✓	✓	✓
• 11-point NRS and VAS scales [39] for:	✓	✓	✓	✓
°Average overall hand pain severity / pain in the most painful joint over the past 2 weeks / 2 days				
• NRS scales for:	✓	✓	✓	✓
°Global disease activity / average thumb pain / average pain in other joints over the past 2days				
°Severity rating of participant nominated main functional problem over the past 2 days				
°Satisfaction with hand function over the past 2 days				
°Hand pain, aching, or stiffness over the past month (no days to all days				
• Global^a^ improvement in hand problem/hand pain/ability to use hands		✓	✓	✓
• Pain elsewhere (pain manikin)	✓			
• Duration of hand pain over the past 12 months (<7 days, 1 to 4 weeks, >1 month, <3 months, >3 months)	✓✓			
• Onset of hand pain (last 12 months, 1 to 5 years, 5 to 10 years, 10 years or more)	✓			
• Quality of life: SF12v2 and OAQoL [40]	✓		✓	✓
• EuroQol EQ-5D [41,42]	✓		✓	✓
• Depression and anxiety: HADS [43]	✓		✓	✓
Demographics and medical history	✓			
Concomitant medication^b^	✓	✓	✓	✓
Adverse events^b^		✓	✓	✓

^a^A six-point Likert scale: completely better, much better, better, no change, worse, much worse. ^b^Also recorded at 1 month and 9 months. AUSCAN, Australian Canadian osteoarthritis hand index; HADS, Hospital Anxiety and Depression Scale; NRS, numerical rating scale; OAQoL, Osteoarthritis Quality of Life Scale; VAS, visual analogue scale.

### Imaging outcome measure

Structural progression between baseline and 12 months will be assessed by calculating change in the Kallman score [44].

### Quality-of-life and health economic outcome measures

Utility will be measured using the EuroQol (EQ-5D-5L), deriving quality-adjusted life years (QALYs) for each participant. Disease-specific quality-of-life (QoL) measures are listed in Table 2.

### Study design

HERO was designed as an investigator-initiated multicentre, randomized, double-blindplacebo-controlled trial to compare the analgesic efficacy of hydroxychloroquine in painful hand OA. Participants will be randomized on a 1:1 basis to hydroxychloroquine or placebo. Given the pragmatic nature of the trial, participants in both treatment arms will be allowed drugs licensed for use in pain management of OA; with drugs and doses determined by clinicians for individual participants. Treatment will be for 12 months.

### Sample size

The sample size for the HERO trial was estimated based on the primary outcome of change in hand pain, between baseline and 6 months, as measured on a 0-to-10 NRS. Mean baseline pain scores of 5.06 (standard deviation (SD), 2.079) and 5.50 (SD 2.5) from previous hand osteoarthritis interventional studies were used to estimate the expected mean (SD) pain scores for this study [45].

The HERO trial is powered to detect an effect size of 0.4, as this is equivalent to the reported effect size of NSAIDs as a treatment for hand OA obtained from two large studies with a total of 654 participants [46,47]. With the estimated mean pain numerical rating scores and SD, in this instance an effect size of 0.4 is equivalent to a 15% change on the pain scale, which lies within the minimal clinically important difference for change in pain in a randomized trial (10 to 20%) [48].

To detect an effect size of 0.4, with 80% power and 5% significance, 101 participants would be required per arm. Allowing for a conservative 20% dropout, a total of 252 participants will therefore need to be recruited into the study.

### Statistical analysis

All analyses will be conducted on an intention-to-treat basis, including all randomized participants in the groups to which they were randomized. Baseline data will be reported descriptively. No formal statistical comparisons of baseline data will be undertaken. The flow of participants through the trial will be presented in a CONSORT diagram. The numbers of participants withdrawing from treatment will be summarized by treatment group.

The primary analysis will estimate the difference in ‘average overall hand pain severity over the previous 2 weeks’ at 6 months between the hydroxychloroquine and placebo groups using a linear mixed model (linear regression for correlated data) adjusting for the baseline measure and other important covariates (for example, chronic drug use).

The secondary analyses will estimate treatment differences at 3 months and 12 months in the same linear mixed model. Other continuous secondary outcomes measured longitudinally will be analyzed using the same methods as those employed for the primary outcome, adjusting for the same covariates as the primary analysis. The ordinal secondary outcomes, measured longitudinally, will be assumed to be continuous and analyzed using the methods described for the primary analysis. Point estimates and their 95% confidence intervals will be presented.

All secondary outcomes will be described descriptively (mean, SD, median, minimum and maximum for continuous data and counts and percentages for categorical data). The SF-12 will be summarized for all components. For each outcome measure the number of nonresponders will be calculated for each treatment group and response rates compared. Appropriate sensitivity analyses will be used to examine the effects of missing data on outcomes.

### Health economics analysis

Health economics evaluation will be carried out to determine the cost effectiveness of using hydroxychloroquine (the intervention) as part of a multi-drug regimen. The intention-to-treat population will be used for all analyses, with resource-use data collected from a National Health Service (NHS) perspective. Medication use will be estimated from the drug-history summary collected during the trial. Health-service use will be measured using a patient health-services utilization questionnaire developed for the HERO trial. Utility will be measured using the EuroQol (EQ-5D-5L), deriving QALYs for each participant. Data on the cost and utility measures will be collected at the same time points as for clinical outcomes. Unit costs of medications will be taken from the British National Formulary. Health-service use cost will be derived from the annual NHS reference cost summary, identifying relevant healthcare resource group codes. Future costs and outcomes will not be discounted, as follow-up in the HERO trial is for 12 months.

The main analysis will be a within-trial analysis. A cost-effectiveness analysis using the primary outcome in the HERO trial, that is cost per unit of reduction in pain score (as measured on a NRS) and cost-utility analysis deriving cost per QALY saved will be conducted. For each analysis, the following summary measures will be estimated:

1. Ratio measure: incremental cost-effectiveness ratio obtained by dividing the incremental cost by the incremental health benefit.

2. Difference measures: net benefit will be calculated based on pre-specified thresholds.

3. Probability measure: a cost-effectiveness acceptability curve will be constructed.

Multiple imputation methods will be used to handle missing data, where needed.

### Trial procedures

Ethical approval for the study has been granted by the Leeds East Research Ethics Committee (reference number 12/YH/0151). Informed written consent will be obtained from all participants.

### Participant recruitment

A total of 252 subjects with symptomatic hand OA will be recruited and randomly allocated to either the treatment or placebo control group. Recruitment methods will include advertisements through the local media and community groups, invitations to previous study participants who have given their consent to be contacted regarding future research projects and liaisons with musculoskeletal physicians, general practitioners and allied health professionals.

### Informed consent and participant confidentiality

Informed consent will be obtained before patients are screened for participation in the HERO trial. The right of the patient to refuse consent without giving reasons will be respected. Further, the patient will remain free to withdraw from the study at any time without giving reasons and without prejudicing any further treatment. The written consent will be obtained by an appropriately delegated clinician who is, by education and experience, qualified to do so, and who has signed and dated the staff authorization and delegation log. The process of obtaining written consent will be clearly documented in the patient’s medical notes. Patient confidentiality will be guaranteed at all times, in line with the requirements of the Data Protection Act and NHS regulations.

### Eligibility criteria

**Figure 2:**
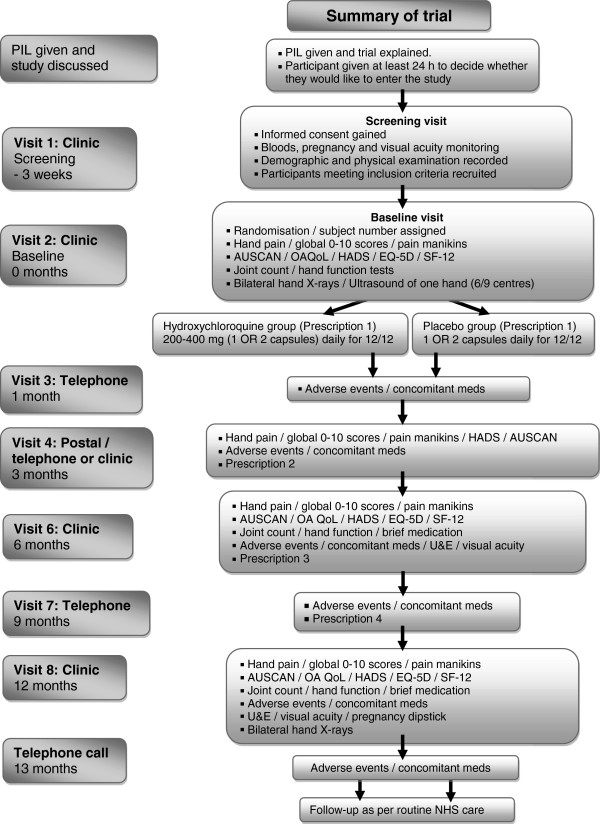
Summary of trial.

Participants must meet the inclusion and exclusion criteria (Table 3) in order to participate. These will be assessed at the screening visit (Figure 2). Potential participants who are deemed ineligible at screening will be allowed a second screening visit if the reason for ineligibility is a temporary status (for example, recent steroid injection; Table 3).

**Table 3:** Eligibility criteria

**Inclusion criteria**	**Exclusion criteria**
Patient-reported inadequate response or toxicity to existing medication (to include paracetamol, oral NSAID or opioid)*	Presence of inflammatory arthritis (for example, gout, reactive arthritis, RA, psoriatic arthritis, seronegative spondylarthropathy, Lyme disease)
	Evidence of plaque psoriasis
Moderately severe symptoms (≥4/10 on a 0 to 10 VAS) at screening	
Symptoms for more than half of days in the last 3 months	OA of the 1st CMC joint and no symptomatic OA in other hand joints
	Oral, IM, IA or IV steroids during the last 2 months*
Fulfil the American College of Rheumatology criteria for OA	Any new hand OA treatment in the previous 2 months, including physiotherapy and provision of new hand splint*
Radiograph of the hands in the past 5 years with changes consistent with OA	Planned hand surgery in the next 6 months
No change in the average weekly dose of analgesics (including NSAIDs) for at least 4 weeks*	Sensitivity, anaphylaxis or allergy to hydroxychloroquine or any other 4-aminoquinoline compound
	Unexplained visual impairment that is not corrected by glasses or presence of any eye problems
Has used chondroitin or glucosamine for at least 4 months with no change to the average weekly dose, is not using or is willing to stop using if recently started*	Pregnant or lactating
	Use of any investigational (unlicensed) drug within 1 month prior to screening or within five half-lives of the investigational agent, whichever is longer*
	Evidence of serious uncontrolled concomitant medical condition, including cardiovascular, nervous system, pulmonary, renal, hepatic, endocrine, GI disease or epilepsy, which in the opinion of the investigator makes them unsuitable for the study
Be able to adhere to the study visit schedule and other protocol requirements	
Capable of giving informed consent, which must be obtained prior to any screening procedures	Uncontrolled disease states, such as moderate or severe asthma or inflammatory bowel disease, where flares are commonly treated with oral or parenteral corticosteroids
	Melanoma or nonskin cancer in the past 3 years*
	IA hyaluronans to the hand joints within the last 6/12*
	Intolerance to lactose
	Significant haematological or biochemical abnormality
	Haemoglobin ≤ 8.5 g/dl
	White cell count ≤ 3.5 × 10^9^/l
	Neutrophils ≤ 1.5 × 10^9^/l
	Platelets ≤ 100 × 10^9^/l
	ALT >2 times ULN for the laboratory conducting the test
	Creatinine >1.5 times ULN for the laboratory conducting the test

*Criteria for which participants may be rescreened if they are ineligible at the initial screening visit. CMC, carpometacarpal; IA, intra-articular; IM, intramuscular; IV, intravenous; RA, rheumatoid arthritis; ULN, upper limit of normal; VAS, visual analogue scale.

### Treatment assignment and allocation concealment

Identical hydroxychloroquine and placebo capsules will be produced to ensure allocation concealment. Upon production, study medication will be packed into numbered bottles according to a randomization schedule. This will be prepared by the contract manufacturer using a computerized random number generator, thereby guaranteeing full allocation concealment. Study medication will be issued in numerical order by the pharmacy. Participants in each site will be randomly assigned to the intervention arm or the placebo arm in a ratio of 1:1 and the randomization will be double-blind. Dosage will be determined as per the schedule in Table 4.

**Table 4:** Dosing schedule

**Male**				**Female**			
**Height**		**Calculated ideal body weight**	**Dosage**	**Height**		**Calculated ideal body weight**	**Dosage**
**cm**	**Feet**			**cm**	**Feet**		
<148 cm	<4^′^10″	<46 kg	200 mg daily	<153 cm	<5^′^0″	<46 kg	200 mg daily
≥148 cm and <166 cm	≥4^′^10″ and <5^′^5″	≥46 and <62 kg	Alternating doses of 200 mg and 400 mg	≥153 and <171 cm	≥5^′^0″ And <5^′^7″	≥46 and <62 kg	Alternating doses of 200 mg and 400 mg
≥166 cm	≥5^′^5″	≥62 kg	400 mg daily	≥171 cm	≥5^′^7″	≥62 kg	400 mg daily

### Blinding

Investigators and participants will remain blinded throughout the trial. Emergency unblinding will be allowed in limited situations that impact on the safety of study participants. Code-break envelopes for the full randomization schedule will be maintained by the Leeds General Infirmary pharmacy, and pharmacies at other sites will hold code-break envelopes for their respective participants.

### Concomitant medications

Investigators will be responsible for the overall management of a participant’s medication, and will ask participants to avoid changing their analgesic or anti-inflammatory medication for the duration of the trial. However, if a participant is experiencing increased pain and requires an increase in the dose of analgesics then the use of paracetamol, topical or oral NSAIDs or opioids, or a combination of these, will be permitted, but the reason for the dose increase, and the dose used, will be documented. Chronic NSAID and opioid use (most days in the last three months) will be included as a covariate in the analysis. Additionally, the following restrictions will apply throughout participant follow-up:

1. Participants will be asked not to use any form of steroids (oral, IV, IA or IM) during the trial period. Any participant requiring oral corticosteroids for any problem will be recorded as a protocol violator. A single articular injection of corticosteroid will be allowed in nonhand joints after the first 6-month phase of the study.

2. Participants will be permitted to continue current use of chondroitin and glucosamine, provided their current dose has been stable for at least four months; however chondroitin or glucosamine therapy will not be commenced during the duration of the trial.

3. Participants will be asked not to start any new nonpharmacological therapies for their hand OA, including physiotherapy and hand splinting.

### Data collection

All data collection will be done on standardized case report forms, which will be completed by site staff, verified by the principal investigator, and returned to the clinical trials unit for data entry. Study sites will also return a patient nonidentifiable participant log and study drug dispensing log to the clinical trials unit. The specific tools used to capture each data element are further detailed next and in Table 2.

### Physical examination and vital signs

Vital signs, height and weight will be performed at screening. Physical examination will also include an examination of both hands to document whether any finger joints are painful, swollen or tender [45,49]. In addition, participants will be asked about visual impairment (not corrected by glasses) at each visit and near visual acuity of each eye (with glasses where appropriate) will be recorded using a standard reading chart at baseline and 12 months [50].

### Medical history and demographic data

Medical history and demographic data, including smoking and alcohol consumption, employment and family history of hand OA, will be recorded.

### Clinical parameters

Self-reported clinical outcomes, including pain and function, quality of life, anxiety and depression will be assessed, as per Table 2.

Handgrip strength will be measured to the nearest pound in both hands using a Jamar dynamometer [51]. Both hands will be alternately assessed in triplicate.

Medication compliance will be measured using the brief medication questionnaire [52,53], and pharmacy pill counts.

### Imaging assessments

Plain radiographs of each hand will be taken at baseline and 12 months (one hand per film), [54]. A posteroanterior (PA) view will be taken, where the palmar aspect of the hand will be placed on the film with the fingers extended, separated slightly and spaced evenly and with the entire forearm placed flat against the X-ray table. A hand map will be provided to each trial site to aid reproducibility of positioning [54] and to ensure consistency of hand positioning between centres.

Radiographs will be scored using the Kallman scale, which scores 24 joints (all but the metacarpophalangeal joints) for six radiological features according to a semi-numerical scale [44,55]. Radiographs will be scored by two readers and the mean score for each feature and the mean total score calculated for analysis.

Baseline ultrasound imaging will be performed for one hand of all participants enrolled at the six centres participating in the substudy. All participants recruited at these sites will have baseline ultrasound imaging of the most painful (or dominant if both equally painful) hand. Each joint will be scored for synovitis and osteophytes. Synovitis will be graded using a semi-quantitative (0 to 3) score using both greyscale (GS) and power Doppler (PD) modalities. [56]. Abnormal PD will be defined as abnormal blood flow located within the synovial hypertrophy; while proliferation within the joint space osteophytes will be scored as being present or absent and will be defined as a ‘step up’ or protrusion of cortical bone seen in more than one plane [57].

Scoring will occur during the acquisition process; however, still images will be taken of all joints for 10 subjects. These will be re-read by the same reader at the end of the study to provide intra-reader reliability for each centre and centrally to provide inter-reader reliability.

### Safety assessments

Adverse events will be recorded throughout the study. Intensity and relationship to the study medication will be ascribed.

### Blood and urine safety assessment

Safety of therapy will be assessed according to the British Society for Rheumatology (BSR) guidelines for hydroxychloroquine [50]:

1. Full blood count (FBC), liver function tests (LFT) and urea, electrolytes (U&E) and creatinine tests will be performed at screening; U&E tests will be repeated at 6 and 12 months for all subjects over 60 or at risk of renal impairment.

2. Urine dipstick pregnancy test at baseline and 12 months for female participants with child-bearing potential.

3. Rheumatoid factor and anti-cyclic citrullinated peptide (CCP) will be measured at baseline.

### Data integrity and management

All data obtained will be kept strictly confidential and will be stored electronically on a database with secured and restricted access. Datasets for each subject will be identified by the participant trial identification number only.

### Withdrawal

Any participant who is unable to tolerate the treatment must be withdrawn from the treatment. The participant will continue to be followed up in the trial. A participant can choose to withdraw from the trial at any time and without giving a reason. However, if a reason is provided this will be recorded. All data will be used up to the point of withdrawal unless the withdrawing participant withdraws consent for use of the data.

### Trial site monitoring

The trial will be overseen and monitored by the York Trials Unit on behalf of the Sponsor, the University of Leeds. Each site will be assessed prior to site setup, and visited again once the fifth participant is recruited or at 20 weeks after the start of recruitment at site, whichever is sooner. The HERO trial was assessed as low-risk by the Medicines and Healthcare Products Regulatory Agency (MHRA), and therefore in addition to the single monitoring visit, procedures for central monitoring at the clinical trials unit have been put in place. This will mainly involve cross-checking logs returned from the research team and site pharmacy for consistency. A data monitoring and ethics committee will provide independent oversight to ensure data quality and compliance with the trial protocol.

### Study site staff training

A centralized introduction and training session was held for all principal investigators and site staff. An ultrasonography training meeting was held separately at which the ultrasound acquisition and scoring protocol was developed. In addition to this, a site initiation visit will be held at all sites, in order to provide specific training to all staff involved in the study ahead of recruitment.

## Discussion

Despite the prevalence of hand OA, there is a distinct lack of robust clinical effectiveness studies into nonsurgical interventions for hand OA [58,59]. Randomized controlled trials (RCTs) of hand OA to date have, in general, been found to be of low quality, with a lack of consistent case definition, a lack of standardized outcome measures and inadequate powering, thereby limiting their interpretation and generalizability to a clinical practice setting. The recent EULAR evidence-based recommendations for the management of hand OA [12], found a notable paucity of clinical trials to guide recommendations for hand OA and highlighted a pressing need for well-designed studies to identify new treatment options.

We have proposed this protocol to determine whether hydroxychloroquine may provide one such treatment option for people with hand OA. There has been growing consensus over the past decade that synovitis plays an important role in the pathogenesis and symptoms of OA [60-62]. This may be reflected in the anecdotal use of the common anti-synovial RA therapies in OA, and the EULAR Recommendations for Hand OA expert consensus group highlighted the need to examine existing slow-acting antirheumatic drugs (SAARDs) for their possible symptomatic and structure-modifying effects in OA. The HERO study therefore aims to examine these effects, as well as to explore the importance of synovitis by including a substudy using ultrasound. Ultrasonography provides more sensitive detection of synovitis than clinical examination [18].

In summary, hand OA is an increasingly prevalent and disabling condition in our ageing society and is already a major cause of healthcare expenditure. Current treatments for hand OA have major limitations and other analgesic treatments are needed. Synovitis is prevalent in OA and previous studies have shown it to be associated with pain in knee and hand OA. Hydroxychloroquine is used in routine practice for treating synovitis in inflammatory arthritides such as RA, is used anecdotally as a treatment for OA and has been shown to be effective at reducing pain in pilot studies. Hydroxychloroquine has an excellent safety profile, with toxicity generally associated with sustained periods of use that, owing to the natural history of hand OA, are unlikely to be an issue. We propose that hydroxychloroquine will be a practical and safe treatment to reduce synovitis and therefore reduce pain in patients with moderate to severe OA hand symptoms. This will potentially provide a new treatment for OA, which could be of particular use in the primary care setting.

## Trial status

Recruitment and follow-up of participants is in progress.

## Abbreviations

ACM: Acetaminophen; AUSCAN: Australian Canadian osteoarthritis hand index; BSR: British Society for Rheumatology; CCRN: Comprehensive Clinical Research Network; CMC: Carpometacarpal; CONSORT: Consolidated Standards of Reporting Trials; EULAR: European League against Rheumatism; FBC: Full blood count; GS: Greyscale; HADS: Hospital Anxiety and Depression Scale; HCQ: Hydroxychloroquine; IA: Intra-articular; IL: Interleukin; IM: Intramuscular; IV: Intravenous; LFT: Liver function test; MeSH: Medical subject headings; MHRA: Medicines and Healthcare Products Regulatory Agency; MRI: Magnetic resonance imaging; NHS: National Health Service; NICE: National Institute for Health and Clinical Excellence; NRS: Numerical rating scale; NSAID: Nonsteroidal anti-inflammatory drug; OA: Osteoarthritis; OA QoL: Osteoarthritis Quality of Life Scale; PA: Posteroanterior; PD: Power Doppler; QALY: Quality-adjusted life year; QoL: Quality of life; RA: Rheumatoid arthritis; RCT: Randomized controlled trial; RHOA: Radiographic hand osteoarthritis; SAARD: Slow-acting antirheumatic drug; SD: Standard deviation; U&E: Urea & electrolytes; ULN: Upper limit of normal; VAS: Visual analogue scale; WOMAC: Western Ontario and McMaster Universities Index of OA.

## Competing interests

The authors declare they have no competing interests.

## Authors’contributions

PGC conceived the study. SRK, PT, JA, NKA, FB, SC, JD, MD, KSD, AG, CEH, TWO, DLS, TLV, RJW, FEW, DJT and PGC participated in its design and coordination, and performed the research. SRK, PT and PGC drafted the manuscript. All authors revised the manuscript and gave final approval of the version to be submitted.
